# Cardiac involvement in MPS patients: incidence and response to therapy in an Italian multicentre study

**DOI:** 10.1186/s13023-022-02396-5

**Published:** 2022-06-29

**Authors:** Simona Sestito, Giada Rinninella, Angelica Rampazzo, Francesca D’Avanzo, Lucia Zampini, Lucia Santoro, Orazio Gabrielli, Agata Fiumara, Rita Barone, Nicola Volpi, Maurizio Scarpa, Rosella Tomanin, Daniela Concolino

**Affiliations:** 1 Pediatric Unit, Department of Medical and Surgical Sciences, Magna Graecia University, Catanzaro, Italy; 2 Laboratory of Diagnosis and Therapy of Lysosomal Disorders, Department of Women’s and Children’s Health, University of Padova, Padua, Italy; 3 Fondazione Istituto di Ricerca Pediatrica Città Della Speranza, Padua, Italy; 4 Division of Pediatrics, Department of Clinical Sciences, Ospedali Riuniti, Presidio Salesi, Polytechnic University of Marche, Ancona, Italy; 5 Department of Clinical and Experimental Medicine, Child Neurology and Psychiatry, University of Catania, Catania, Italy; 6 Department of Life Sciences, University of Modena and Reggio Emilia, Modena, Italy; 7 MetabERN, Regional Coordinating Center for Rare Diseases, Udine University Hospital, Udine, Italy; 8 Pediatric Unit, Department of Science of Health, Magna Graecia University, Catanzaro, Italy

**Keywords:** Mucopolysaccharidoses (MPS), Glycosaminoglycans (GAGs), Heart involvement, Valvulopathies, Enzyme replacement therapy (ERT**)**

## Abstract

**Background:**

Mucopolysaccharidoses (MPSs) are a group of lysosomal storage disorders caused by the deficit of lysosomal hydrolases involved in the degradation of glycosaminoglycans (GAGs). The course is chronic and progressive, with multisystemic involvement that often leads to cardiovascular disease. We describe the overall incidence and type of cardiac damage in a cohort of Italian MPS patients, and their progression over time, also with reference to treatment efficacy in patients under Enzyme Replacement Therapy (ERT). Moreover, we report a possible association between genetic variants and cardiac phenotype in homozygous and hemizygous patients to understand whether a more aggressive clinical phenotype would predict a greater cardiac damage.

**Results:**

Our findings confirm that cardiac involvement is very common, already at diagnosis, in MPS VI (85.7% of our cohort), and in MPS II (68% of our cohort) followed by MPS I subjects (55% of our cohort). The most frequent heart defect observed in each MPS and at any time-point of evaluation was mitral insufficiency; 37% of our patients had mitral insufficiency already at diagnosis, and 60% at post-ERT follow-up. After at least six years of treatment, we observed in some cases (including 6 MPS II, 2 MPS IV and 2 MPS VI) a total regression or improvement of some signs of the cardiac pathology, including some valve defects, though excluding aortic insufficiency, the only valvulopathy for which no regression was found despite ERT. The general clinical phenotype proved not to be strictly correlated with the cardiac one, in fact in some cases patients with an attenuated phenotype developed more severe heart damage than patients with severe phenotype.

**Conclusions:**

In conclusion, our analysis confirms the wide presence of cardiopathies, at different extent, in the MPS population. Since cardiac pathology is the main cause of death in many MPS subtypes, it is necessary to raise awareness among cardiologists about early cardiac morpho-structural abnormalities. The encouraging data regarding the long-term effects of ERT, also on heart damage, underlines the importance of an early diagnosis and timely start of ERT.

## Background

Mucopolysaccharidoses (MPSs) are rare monogenic metabolic disorders due to the deficit of lysosomal enzymes involved in the degradation of glycosaminoglycans (GAGs), with consequent substrate accumulation in various cell types and tissues, and progressive multi-organ dysfunction. Eight distinct subtypes of MPS (I, II, III, IV, VI, VII, IX and X), the result of 12 enzyme deficiencies, have been described. All MPSs are inherited in an autosomal recessive manner except MPS II, which follows an X-linked inheritance pattern. The incidence of MPSs varies within each disorder and in different populations and ethnic groups, with a global prevalence between 1.9 and 4.5:100,000 live births [1]. Patients with MPS generally appear normal at birth, but later on they may develop multiple clinical manifestations including coarse facial features, skeletal abnormalities leading to growth impairment, poor joint range of motion, organomegaly, corneal clouding, hearing loss, valvular heart disease, cardiac hypertrophy. Also neurological involvement is present in MPS IH (Hurler), in the severe forms of MPS II and VII, in most MPS III patients [2, 3]. MPS diagnosis proceeds from clinical suspicion, going through biochemical analysis, including urinary GAGs and enzymatic assays, and finally is confirmed by molecular diagnosis. Beyond symptomatic therapy, substantial progress has been made in the last years in understanding the pathophysiology of lysosomal storage disorders, leading to newly targeted therapeutic options [4]. Hematopoietic stem cell transplantation (HSCT) has been successfully applied almost exclusively in Hurler syndrome and rarely in other MPSs [5], and the best results were obtained when it was performed before the age of two [1, 6–8]. The observation that genistein considerably inhibits the synthesis of GAGs in cultured fibroblasts [9], at some extent also registered in the MPS II mouse model [10], suggested the use of this molecule as substrate reduction therapy for these patients [11], although its clinical efficacy remains uncertain to date. Enzyme replacement therapy (ERT) is currently available for five types of MPS (MPS I, MPS II, MPS IVA, MPS VI, MPS VII). It has been widely demonstrated that ERT is effective in reducing urinary GAG excretion, mostly resolving hepatosplenomegaly and improving cardiopulmonary function [12, 13]; moreover, ERT proved to be safe, so that it is currently practicable in home setting for some MPS subtypes [14]. Gene therapy was also considered as a therapeutic option for MPSs [15], however its application in clinical practice is still rare.

One of the most important clinical aspects of MPSs is cardiovascular pathology, which is frequent and with a wide spectrum of manifestations [16, 17]. Cardiac abnormalities occur in all MPS subtypes, with the most common being valvular defects and cardiac hypertrophy, that are the result of GAG accumulation in the spongiosa of cardiac valves, myointima of coronary arteries and myocardium [2]. The onset and extent of cardiovascular involvement differs for each MPS type, however cardiovascular system impairment significantly increases morbidity and mortality in affected patients [18, 19].

In this paper, we describe the overall incidence and characteristics of cardiac damage in a cohort of MPS patients enrolled in a multicentre, retrospective Italian survey.

The aim of the study was to assess which and how many cardiac defects were present since diagnosis, overall and for each type of MPS, in order to understand if any pathological cardiac signs in a suspected patient could help to address the correct diagnosis. We also evaluated the progression over time of the heart involvement, also in reference to treatment efficacy in patients under ERT. Finally, we searched possible associations between each variant and the cardiac phenotype in homozygous and hemizygous patients, to understand whether a more severe clinical phenotype necessarily predicts a more serious cardiac damage.

## Results

### General characteristic of the population enrolled

Sixty patients, 37 males and 23 females, age range 3–51 years at the time of enrolment, affected by 5 different MPS subtypes (9 patients with MPS I; 16 with MPS II; 8 with MPS IIIA; 5 with MPS IIIB; 1 with MPS IIID; 12 with MPS IVA; 2 with MPS IVB; 7 with MPS VI) were enrolled in the study.

The overall mean age at diagnosis was around 5 years, with the minimum age of 4 months in a MPS I subject, attributable to a known familiarity for the pathology (commonly, in fact, clinical symptomatology is not present so early). The maximum age of 44 years was instead reported in MPS II, for a patient presenting a very attenuated non-neuropathic phenotype. By relating the diagnostic timing to the type of MPS, we observed that the peak of diagnoses occurred between the 1st and the 3rd year of life for MPS II (median = 2.85 y), MPS IV (median = 2.75 y) and MPS VI (median = 1.8 y). For MPS I we found two peaks, one within the 1st year of life, probably due to the early signs present in some patients, and the other between the 3rd and the 10th year, referable to more attenuated forms of disease (overall median = 4.0 y). As for MPS III, the apex of diagnoses was reached between the 3rd and the 10th year of life, probably due to the progressive nature of the disease and to non-specific neurological symptoms exhibited by these patients, which made difficult the differential diagnosis with other neurodegenerative diseases. Specifically, median value was 5.3 and 6.0 years for MPS IIIA and MPS IIIB, respectively.

Regarding treatment, data collected showed that: 4 patients (7%) (3 with MPS I and 1 with MPS II) had received hematopoietic stem cell transplantation, 5 patients (8%) with MPS III were treated with genistein (substrate reduction therapy), 40 patients (67%) (6 patients with MPS I, 15 with MPS II, 12 with MPS IVA, 7 with MPS VI) were on ERT, one of which had previously received HSCT. For eleven patients (18%) (9 affected by MPS III and 2 by MPS IVB) no treatment was available.

As for ERT, the most common treatment applied, Table 1 shows average values (in years) of the time elapsed between diagnosis and initiation of ERT, and of the age at start of ERT for each MPS. Considering the forty patients on ERT, the average time elapsed between diagnosis and start of treatment was 5 years and 10 months (median 3 years), while the mean age at start of ERT was 10 years and 5 months (median 7 years and 4 months).

**Table 1:** Main characteristics of the MPS patients enrolled in the present study

MPS subtype	Number of pts (male; female)	Clinical phenotype	Age at diagnosis (years)^§^	Time between diagnosis and start of ERT (years)^§^	Age at start of ERT (years)^§^
MPS I	9 (6 M; 3F)	6 non-neuropathic, 3 neuropathic	2.5 ± 2^#^	1.5 ± 1.8	6.2 ± 6
MPS II	16 (15 M; 1F)	6 non-neuropathic, 11 neuropathic	4.6 ± 3*	4.6 ± 8	10.5 ± 10*
MPS IIIA	8 (5 M; 3F)	1 mild, 7 severe	6.27 ± 5	–	–
MPS IIIB	5 (2 M;3F)	5 severe	4.28 ± 3	–	–
MPS IIID	1 (1 M; 0F)	1 severe	5.48	–	–
MPS IVA	12 (4 M; 8F)	5 slowly progressing,7 rapidly progressing	3.3 ± 2	9.5 ± 9.9	12.8 ± 10
MPS IVB	2 (2 M; 0F)	2 slowly progressing	10 ± 3	–	–
MPS VI	7 (2 M; 5F)	2 slowly progressing, 5 rapidly progressing	2 ± 0.7	4.7 ± 3	6.7 ± 3

^#^One patient, diagnosticated at fourteen years of age, was excluded from the average. *One patient, diagnosticated at forty-four years of age, was excluded from the average; ^**§**^data expressed as mean ± standard deviation

### Description and evolution of cardiac pathology in the population examined in the three time-points considered

Data analysis evidenced that 32 out of 60 patients (53%) showed cardiac involvement at the time of diagnosis and 50 out of 60 (83%) at T1, the time at enrolment in this study.

**Fig. 1:**
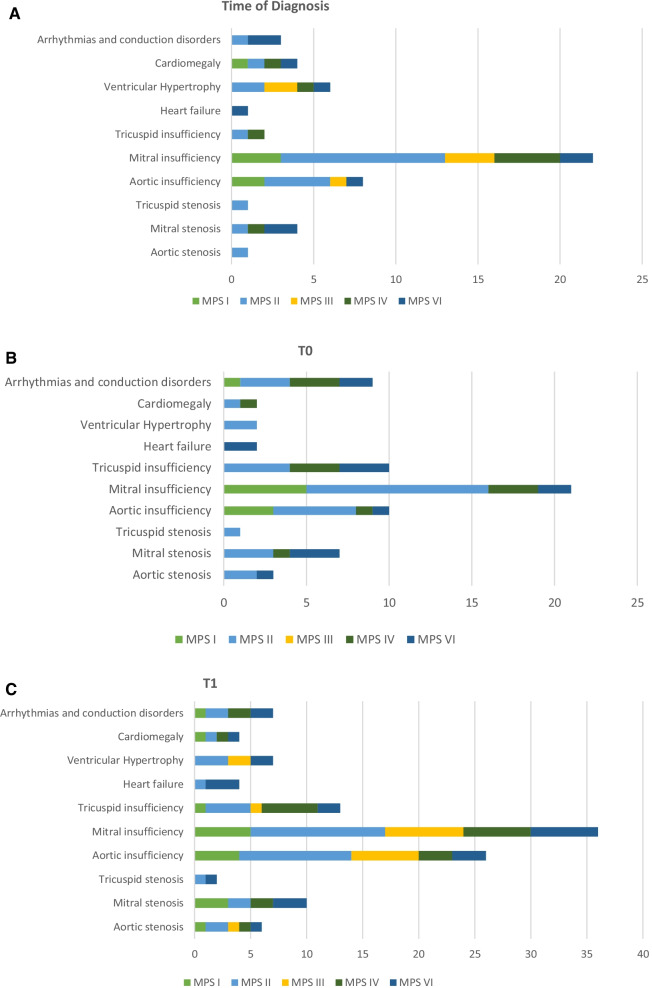
(**A**) Cardiac involvement at the time of diagnosis based on the MPS subtype. (**B**) Cardiac involvement at T0 based on the MPS subtype. (**C**) Cardiac involvement at T1 based on the MPS subtype

Figure 1a shows cardiac involvement found in the cohort of patients at diagnosis, grouped by MPS subtype. At diagnosis (TD) cardiac involvement was a very common finding in patients with MPS VI (85.7% of the patients) and MPS II (68% of the patients), followed by MPS I (55% of the patients). Overall, 37% of all examined patients had mitral insufficiency, 13% had aortic insufficiency, 10% had ventricular hypertrophy at TD, while the other cardiac manifestations presented with a lower incidence.

It is interesting to highlight the relationship between the age of the patients at the time of diagnosis and their cardiac involvement. Out of the 6 patients diagnosed within the first year of life, 2 had cardiac involvement at diagnosis: a patient with a classical form of MPS IVA, who had only a mild tricuspid insufficiency and a patient with a rapidly progressing form of MPS VI, who presented aortic insufficiency and mild mitral stenosis. Out of 24 patients diagnosed between the 1st and the 3rd year of age, 14 had cardiac disease at the time of diagnosis, including a patient with MPS IIIB. Particularly, it is striking to observe that out of the 5 patients with MPS VI diagnosed in this age group, 4 (80%) already presented cardiac pathology at TD. Out of 24 patients diagnosed between the 3rd and the 10th year of age, 15 presented heart disease at TD; all MPS I patients diagnosed in this age group already showed cardiac involvement at the time of diagnosis; the only patient with MPS VI in this group, with a mild clinical phenotype, had moderate mitral insufficiency already at TD. Out of the 6 patients diagnosed over 10 years of age, 3 had heart disease at TD; five patients presented an attenuated clinical phenotype and there was one MPS IIIB patient with a severe phenotype who did not present any cardiac diseases at TD.

Figure 1b shows cardiac involvement found in the cohort of patients at T0, the closest time point before the start of therapy, in relation to the MPS subtype. As previously mentioned, the clinical evaluation at this time point was not possible for MPS III and MPS IVB patients, for which no therapy was available. At T0, the incidence of cardiac involvement increased especially among patients affected by MPS II (87.5% of MPS II patients) and by MPS IVA (50% of MPS IVA patients). In particular, we recorded an increased incidence with respect to TD, for tricuspid insufficiency (from 3% of patients at TD to 17% of patients at T0), for conduction disorders (from 5 to 15%) and for mitral stenosis (from 6% to 11.7%) while the incidence of other cardiac disorders remained almost unchanged.

**Fig. 2:**
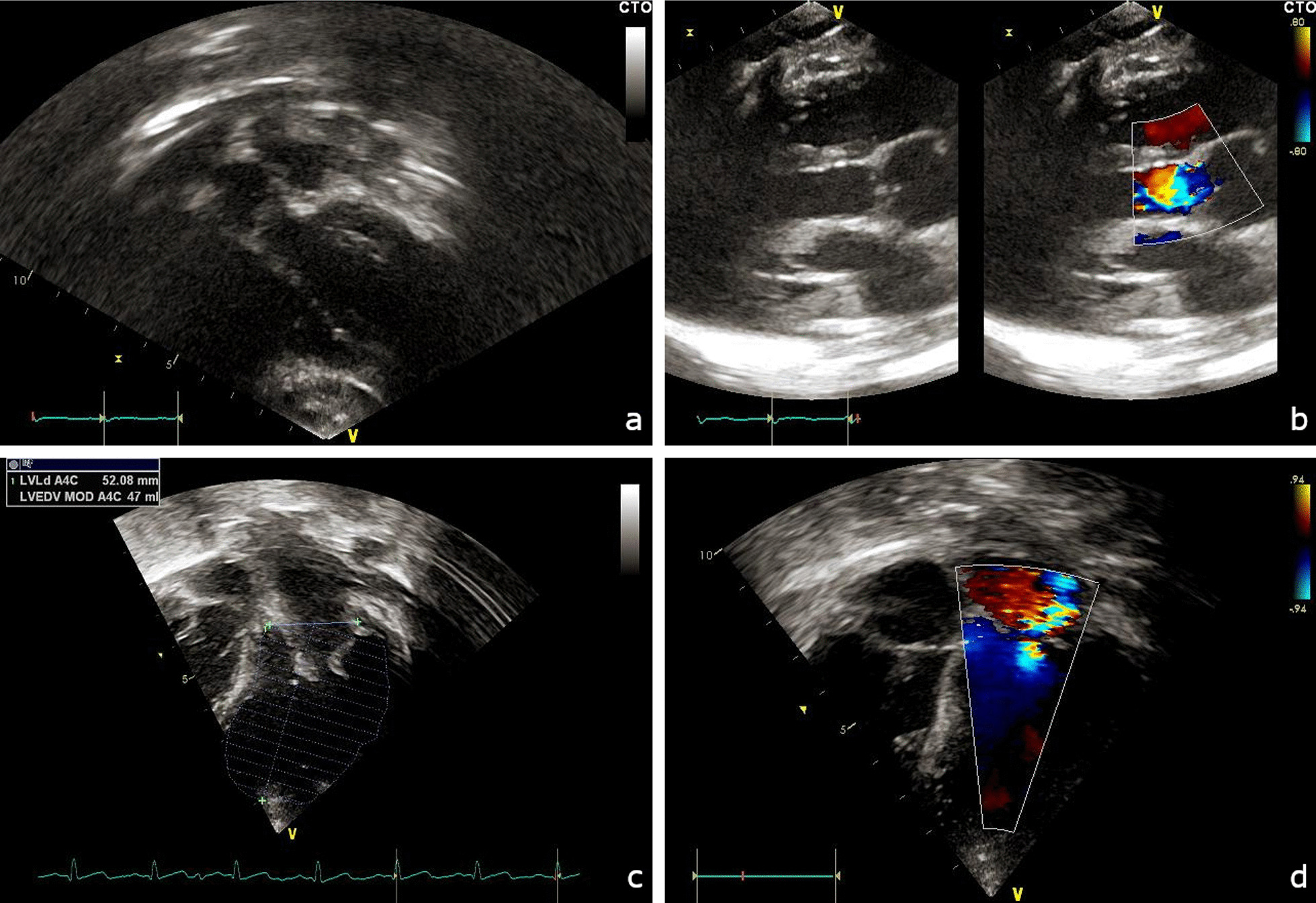
Representative echocardiographic images from two MPS VI patients. Aortic valve insufficiency in colour flow from apical four chambers view (**a**) and parasternal long axis (**b**), evidencing the thickness of valvular leaflets. Mitral valve in apical four chambers (**c**), showing mixomatous leaflets, and colour jet (**d**) evidencing a moderate-severe valve insufficiency

Figure 1c shows the cardiac involvement reported in the cohort of patients at T1, the time of enrolment in this study, with reference to the MPS subtype. At T1, the incidence of cardiac diseases in our population increased for each MPS subtype, especially for MPS III patients (from 35.7% at TD to 71% at T1) and for MPS I patients (from 55% at T0 to 78% at T1), including almost all subjects in the case of MPS II and MPS VI (94% and 100%, respectively). At T1 we recorded an increased incidence especially for mitral insufficiency (from 37% of patients at T0 to 60% of patients at T1) and for aortic insufficiency (from 17% of patients at T0 to 43.3% of patients at T1). Figure 2 shows representative echocardiographic images evidencing mitral and aortic insufficiencies in two MPS VI patients under ERT.

Considering the entire population under examination, mitral insufficiency was the most frequently encountered cardiac pathology, both at diagnosis (Fig. 1A) and at follow-up times (Fig. 1B, C), in all MPS subtypes. The second most frequent cardiac pathology was aortic insufficiency. Mitral stenosis, tricuspid insufficiency, aortic stenosis, ventricular hypertrophy, heart failure, conduction disorders and cardiomegaly were less frequently registered. Arrhythmias were rare and there were no cases of congenital cardiac abnormalities requiring cardiac surgery. In general, along with disease progression there was a substantial increase in aortic insufficiency and mitral insufficiency at T1, compared to the time at diagnosis, as well as an increased incidence of other cardiac pathologies, confirming the degenerative nature of this group of metabolic diseases. Multiple heart manifestations were frequently found in a single subject. The most common comorbid cardiac disorders were mitral insufficiency or steno-insufficiency and aortic insufficiency, sometimes even associated with aortic stenosis. Ventricular hypertrophy, or more correctly pseudohypertrophy, was often associated with valvular defects of the left heart but it did not correlate with the heart failure. Heart failure, instead, was often associated with severe mitral stenosis and aortic steno-insufficiency, as well as with conduction disorders.

### Description and evolution of cardiac pathology in the different MPSs

Among MPS I valvular disorders the most frequent were mitral insufficiency, aortic insufficiency, mitral stenosis, aortic stenosis and tricuspid insufficiency, ordered by incidence. Cardiomegaly and conduction disorders were rare. For MPS I there was no regression of valve damage either in patients undergoing ERT or in those receiving HSCT. Only one patient, who received HSCT just before the age of 11, showed complete regression of cardiomegaly, but with no effects on valvular disease.

The highest incidence of mitral insufficiency occurred in MPS II, along with a large spectrum of valvular defects, conduction abnormalities, arrhythmias and ventricular pseudohypertrophy. In 3 MPS II patients, including 2 with neuropathic and 1 with non-neuropathic phenotype, we registered a total regression of mild tricuspid insufficiency approximately 6 years after the beginning of ERT (T1). In 3 other MPS II subjects, 2 with neuropathic and 1 with non-neuropathic phenotype, a total regression of mild mitral insufficiency was documented in the assessment conducted approximately 6 years after the start of ERT. Of the remaining MPS II patients (N = 10), only one received HSCT at about 3 years of age and developed only mild aortic regurgitation at T1 (about 20 years after transplantation). In the other 9 patients, the heart damage found at diagnosis, remained stable during the follow-up or worsened despite ERT. For 7 of these 9 patients, the T1 assessment was conducted within 6 years from the start of ERT.

In MPS III, as expected, cardiac involvement was present, but it was less heterogeneous, essentially showing mitral and aortic insufficiency. Of the 14 MPS III subjects, only 5 presented heart disease at diagnosis, while the incidence doubled at T1 (10/14), with progressive worsening of the valve disease. The remaining 4 patients did not develop any heart damage.

In MPS IVA, we found the highest number of patients with right valvular defects, specifically tricuspid insufficiency (4 out of 12 examined subjects). In 2 MPS IVA patients, examined at T1 less than 1 year after the start of ERT, a correction of the arrhythmias, present at diagnosis, was observed together with the reduction of ventricular hypertrophy in one case and cardiomegaly in the other one. In all other patients, heart damage remained stable or worsened. This is probably due to the fact that ERT for this type of MPS has only been available for a few years and therefore in many cases the assessment at the time of enrolment (T1) was too close to the start of therapy. Of the 2 patients with MPS IVB, both with slowly progressive phenotype, only one developed valvular heart disease that worsened over time, as no therapy was available.

MPS VI patients showed a wide variety of cardiac disorders. Compared to other MPSs, heart failure was more frequent in these patients. Of the 7 MPS VI subjects examined, the only one with a mild clinical phenotype showed a reduction in the degree of mitral insufficiency after about 6 years from the start of ERT, however developing mild aortic insufficiency during the follow-up. In a patient with a severe phenotype, there was a total regression of moderate tricuspid insufficiency and mild heart failure at evaluation, about 10 years after the start of ERT. However, he developed severe tricuspid stenosis. In the other cases of MPS VI, heart damage, especially valve damage, worsened over time.

Considering the entire population under examination, for aortic insufficiency we could not find any degree of regression in spite of the therapy. At T1, it was, indeed, often recorded an appearance or an aggravation of aortic regurgitation, with an incidence rate, which doubled from the time of diagnosis to the enrolment in this study. At time T0, pre-therapy, we saw an incidence rate of tricuspid insufficiency almost comparable to that of aortic insufficiency. After the start of therapy, the incidence of aortic insufficiency increased significantly while the same did not happen for tricuspid insufficiency, the incidence of which slightly increased.

Regarding heart involvement following HSCT, we could not see any positive outcome of the treatment in preventing or stopping the progression of valve damage in any of the 4 transplanted patients.

Considering the 40 patients who underwent ERT, the incidence of tricuspid insufficiency was 10/40 (25%), both at T0 and T1, while the incidence of aortic insufficiency doubled from 20% (8/40) at T0 to 40% (16/40) at T1. For the most frequent sign, i.e. mitral insufficiency, in patients under ERT, the incidence was 11/40 (27.5%) at T0 and 16/40 (40%) at T1. Thus, the increase in incidence following therapy was slightly lower for mitral insufficiency compared with aortic insufficiency.

### Cardiac damage in relation to ERT starting age

**Fig. 3:**
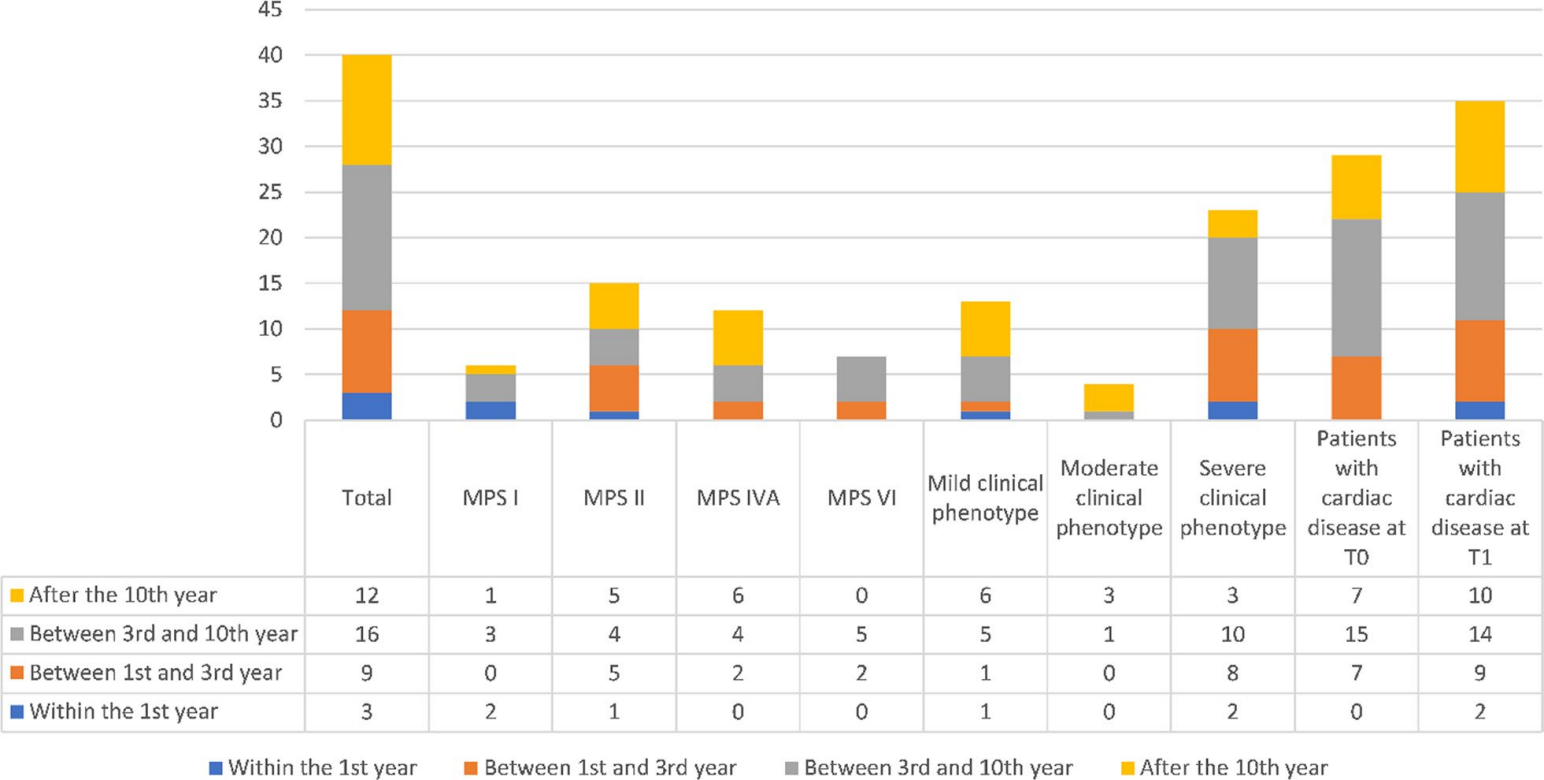
Patients undergoing ERT clustered by age at the start of ERT treatment (n = 40)

Figure 3 shows patients in ERT grouped by age at start of treatment, analysed as MPS subtype, clinical phenotype and the presence of cardiac damage at T0 and T1. Only 3 patients (2 MPS I and 1 MPS II) started ERT within their first year of life and, two of them, a MPS I with Hurler-Scheie phenotype and a MPS II with neuropathic phenotype, developed heart damage. Specifically, the MPS I patient developed mild mitral and tricuspid insufficiency, while the MPS II patient, showed only conduction disorders at ECG. Both patients did not develop severe heart disease, possibly suggesting that a very early ERT administration may not prevent heart damage, though it might mitigate its progression.

Nine patients started ERT between the first and the third year of life, and 8 of them (89%) developed a severe clinical phenotype. The one developing a non-neuropathic phenotype was a MPS II subject showing at diagnosis mild aortic and mitral insufficiencies, which completely regressed at T1. Two patients with severe phenotype, showed cardiac involvement only at T1: a MPS II patient presenting mild aortic stenosis and mild mitral insufficiency; and a MPS VI patient developing in few years a moderate mitral insufficiency and a left ventricular hypertrophy.

Most of the patients under examination started ERT between the 3rd and the 10th year of life and 10 of them (62.5%) presented with a severe clinical phenotype. This data should be analysed considering that for many of these patients ERT was unavailable for some years after diagnosis. Thirteen patients out of 16 (81%) showed cardiac involvement at diagnosis and this percentage already increased at T0 (94%). In only one patient, presenting a neuropathic form of MPS II, a regression of the mild mitral regurgitation reported at diagnosis, was observed at T1, approximately 6 years after the start of ERT. Six patients (37,5%) showed a stable heterogeneous and moderate/severe cardiac involvement.

Of the 12 patients who started ERT after 10 years of age, 75% (9/12) had a mild/moderate clinical phenotype. Despite this, 7 of them showed cardiac involvement before the start of treatment, including 5 MPS II patients, one with a neuropathic phenotype and 4 with an attenuated phenotype. Only 2 patients, a MPS I and a MPS IVA, did not develop any cardiac involvement. In 5 (4 MPS II and 1 MPS IVA) of these 12 patients (42%) a moderate valvular heart damage was found, and in the whole cluster following ERT there was no regression, but rather a worsening, of pre-existing heart diseases, even in patients with mild phenotype.

By evaluating how therapy could influence severity and progression of each cardiac defect, we found that the most frequent cardiac pathology was mitral insufficiency, regardless to the start of ERT or the length of treatment. The incidence of aortic insufficiency, on the other hand, increased with increasing age at start of treatment. Ventricular hypertrophy and conduction disorders appeared more diffused in patients treated before 3 years of age; however, we interpreted this data in the light of a more severe phenotype that led to earlier diagnosis and therapy.

### Cardiac phenotype and genomic variants

Molecular diagnosis was available for 50 of the 60 patients enrolled (83%), and previously described [20]. Table 2 shows the genetic variants found either in homozygosis (HO) or in hemizygosis (HE) (for MPS II) and the related clinical phenotypes, with particular reference to cardiac involvement, this allowing to possibly evaluate the effect of each single mutation on the patient cardiac phenotype. In a few cases, our data was also compared to cardiac data reported in literature, for patients carrying the same variants.

**Table 2:** List of variants identified in hemyzygotic and homozygotic patients and related cardiac phenotype

Gene	Variant	Type of mutation	Residual enzyme activity	Clinical phenotype	Cardiac phenotype at T1	Type of treatment
*IDS*	c.187A > G p.Asn63Asp	Missense	NA	Non-neuropathic	Moderate aortic insufficiency and mild mitral insufficiency	ERT
*IDS*	c.1264 T > C p.Cys422Arg	Missense	NA	Non-neuropathic	Aortic, mitral and tricuspid insufficiencies and cardiomegaly	ERT
*IDS*	homologous recombination *IDS-IDS2*	Homologous recombination	0.69%	Neuropathic	Regression of the mild mitral insufficiency present before starting therapy	ERT
*IDS*	c.811A > T p.Arg271Trp	Missense	0.69%	Neuropathic	Mild aortic and mitral insufficiencies	ERT
*IDS*	c.589_592del; p. Pro197Thrfs*15	Deletion	3.3%	Neuropathic	Hypertrophy and mild mitral insufficiencies	ERT
*IDS*	c.1403G > A p.Arg468Gln	Missense	0.94%	Neuropathic	Mild aortic insufficiency	ERT
*IDS*	c.1400C > T p.Pro467Leu	Missense	5%	Neuropathic	Mild mitral and tricuspid stenosis, mild aortic insufficiency, moderate mitral insufficiency and grade 1 heart failure	ERT
*IDS*	del ex 1–7 two deletions in tandem with 2 duplications located 1.2 Mb distally from *IDS*	Deletion	0.72%	Neuropathic	Mild aortic stenosis and mild mitral insufficiency	ERT
*IDS*	c.708G > A p.Lys236Lys	Sense	2.6%	Non-neuropathic	Mild aortic insufficiencies	ERT
*IDS*	c.592G > A p.Asp198Asn	Missense	NA	Neuropathic	Mild mitral insufficiency	ERT
*IDS*	c.1478G > C p.Arg493Pro	Missense	Absent	Neuropathic	Mild mitral insufficiency	ERT
*IDS*	c.359C > G p.Pro120Arg	Missense	2.6%	Neuropathic	Mild aortic and tricuspid insufficiencies, moderate mitral insufficiencies	ERT
*IDS*	c.1563A > T p.Glu521Asp	Missense	Absent	Non-neuropathic	Hypertrophy, moderate aortic and mitral steno-insufficiency (diagnosis at 44 years)	ERT
*IDS*	deletion of the whole *IDS* gene	Deletion	Absent	Neuropathic	Cardiac impulse conduction disorders	ERT
*SGSH*	c.544C > T p.Arg182Cys	Missense	6%	Severe	Absent	Not on treatment
*SGSH*	c.617G > C p.Arg206Pro	Missense	17.2%	Mild	Absent	Not on treatment
*SGSH*	c.197C > G p.Ser66Trp	Missense	Heavily reduced	Severe	Mild aortic insufficiency	Genistein
*SGSH*	c.220C > T p.Arg74Cys	Missense	Absent	Severe	Moderate aortic and mild mitral insufficiencies	Not on treatment
*GNS*	c.814C > T p.Gln272*	Nonsense	0.45%	Severe	Absent	Not on treatment
*GALNS*	c.29G > A p.Trp10*	Nonsense	9.7%	Rapidly progressing	Grade 2 heart failure and moderate aortic insufficiency	ERT
*GALNS*	c.29G > A p.Try10*	Nonsense	3.2%	Slowly progressing	Absent	ERT
*GALNS*	c.1519 T > C p.Cys507Arg	Missense	13.6%	Rapidly progressing	Mild mitral stenosis	ERT
*GALNS*	c.1520G > T p.Cys507Phe	Missense	10.8%	Rapidly progressing	Mild tricuspid insufficiencies	ERT
*GALNS*	c.1520G > T p.Cys507Phe	Missense	5%	Rapidly progressing	Mild tricuspid insufficiency	ERT
*GALNS*	c.1043 C > A p.Thr348Asn	Missense	5.5%	Rapidly progressing	Mild mitral insufficiencies	ERT
*ARSB*	c.1213 + 6 T > C –	Insertion	Absent	Rapidly progressing	moderate mitral and mild tricuspid insufficiencies	ERT
*ARSB*	c.944G > A p.Arg315Gly	Missense	Absent	Rapidly progressing	Severe tricuspid stenosis and mild mitral insufficiency	ERT
*ARSB*	(898 + 1_899-1)–(1142 + 1_1143-1) del	Deletion	20.4%	Rapidly progressing	Heart failure, mild mitral steno-insufficiency, cardiac impulse conduction disorders	ERT
*ARSB*	(898 + 1_899-1)–(1142 + 1_1143-1) del	Deletion	Absent	Rapidly progressing	Heart failure NYHA 1, ventricular hypertrophy, severe aortic stenosis, moderate mitral stenosis and mild aortic insufficiency	ERT
*ARSB*	c.323G > T p.Gly108Val	Missense	50.1%	Rapidly progressing	Ventricular hypertrophy and moderate mitral insufficiency	ERT
*ARSB*	c.245 T > C p. (Leu82Pro)	Missense	18%	Slowly progressing	Heart failure NYHA 1, mild mitral stenosis, moderate aortic, mitral and tricuspid insufficiency, cardiac impulse conduction disorders	ERT

*HE* hemizygous, *HO* homozygous, *NA* Not Available

Thirty-one patients were carrying 28 unique disease-causing variants, either in HO or HE condition, since 3 variants were present each in 2 patients; 19 out of 28 were missense variants, 4 were deletions (2 small and 2 large), 2 were nonsense, together with a sense variant, a homologous recombination and a splicing site variant. All genetic variants were previously reported by Zanetti et al., including 4 novel mutations for which an in silico prediction of pathogenicity could be performed, as reported in [20]. Here we examined whether the type of mutation could be related to the cardiac involvement. This specific evaluation was not conducted for MPS I since no homozygous patients were present, while we could examine all MPS II, 5 MPS III, 6 MPS IVA and 6 MPS VI patients, for a total of 31 subjects and 28 variants. Among the mutations associated to a *severe phenotype* in our patient cohort (21/28 = 75%), only 10 had been previously reported in literature. For 4 of them the cardiac phenotype was available and only in one case, related to *GNS* variant c. 814C > T, our findings were consistent with the previous report. The deletion in the *ARSB* gene c. [898 + 1_899-1] _ [1142 + 1_1143-1] del, detected in our cohort in 2 unrelated patients with significant cardiac involvement, was described in a patient with mild cardiac phenotype in a previous study [21]. The *IDS* missense variant c.1403G > A, reported in our study in a patient with mild aortic insufficiency, was previously described in literature in a patient presenting a severe cardiac involvement. Hence, from this data we can conclude that there is no association between molecular genetic data and severity of cardiac phenotype.

Overall, 4 patients did not present any cardiac involvement, 2 patients with a severe and 2 patients with a mild clinical phenotype. Thirteen of the 23 severe patients (56.5%) developed a mild to moderate heart disease, mainly with valvular damage. Only two patients with a non-neuropathic MPS II clinical phenotype had severe cardiovascular involvement. One of them presented a new variant of the *IDS* gene: c.1563A > T (p.Glu521Asp), hence the comparison with the previous literature was not possible; in addition, the ventricular hypertrophy and the mitral steno-insufficiency were possibly to be ascribed to the late age at diagnosis (44 years). The other patient, carrying the variant c.1264 T > C (p.Cys422Arg), presented aortic, mitral and tricuspid valves insufficiencies and cardiomegaly. This variant correlated with a neuropathic clinical phenotype in previous studies, where the description of heart involvement had not been conducted [22]. Out of the 3 variants identified each in 2 patients, 2 were detected in unrelated subjects: the *GALNS* variant c.29G > A and the *ARSB* variant (898 + 1_899-1)_(1142 + 1_1143-1) del. As for the former, the 2 patients presented a totally different cardiac phenotype (severe and absent respectively), while for the latter both patients showed a severe cardiac involvement. The variant c.814C > T (p.Gln272*) carried on the *GNS* gene by a patient with MPS IIID, who had no cardiac involvement throughout our entire follow-up, had previously been described in another homozygous patient, whose cardiac phenotype, following examination including ECG, was reported as normal [23].

## Discussion

One of the aims of the present study was to describe and analyse the incidence of cardiac involvement in a large group of patients (n = 60) with MPS, enrolled in a multicentre program.

Our findings confirm what emerges from literature: cardiovascular pathology prevails in the MPS subtypes that involve a greater accumulation of dermatan-sulphate due to enzyme deficits [2]. Particularly, cardiac involvement is a very common finding in MPS II and in MPS VI, followed by MPS I. It turned out that the most frequent heart defect for each type of MPS and at any time of evaluation was mitral insufficiency. As many as 37% of patients had mitral insufficiency already at diagnosis and 45% of these were MPS II. Therefore, we can conclude that valve disorders, particularly mitral insufficiency, should be sought already at diagnosis in these patients, and that, in some cases, cardiac disorders together with a suspected clinical phenotype can support the diagnosis of MPS. As regards age at diagnosis, 6 of 7 MPS VI patients had cardiac involvement at diagnosis and 5 of them were younger than 3 years old. This highlights how cardiological involvement can be evident even at an early age, especially in some subtypes of MPS.

Regarding the efficacy of ERT on heart damage, we observed that in patients who started ERT within the first year of age, heart involvement, although present, is often milder. Based on the results obtained, the hypothesis that ERT is not able to prevent valve damage is confirmed, since heart valves are avascular connective tissue and the therapeutic enzyme, indeed, is distributed by systemic perfusion; therefore, the amount of enzyme that reaches the avascular tissue may be limited. However, data in literature shows that the cardiac damage could be prevented with ERT, as long as it is started very early in life, before the fibrosis of the valve leaflets is established [24, 25]. Early diagnosis and treatment, therefore, play a role of primary importance, and, given the availability of ERT for five MPSs (MPS I, MPS II, MPS IVA, MPS VI, MPS VII), it would be desirable to achieve early diagnosis, hopefully in a presymptomatic phase, before organ damage has occurred, for example through the establishment of neonatal screening programs.

Another aspect here investigated was the effect of long-term ERT on heart damage. We observed in 10 cases (6 MPS II, 2 MPS IV and 2 MPS VI) a regression or improvement of cardiac symptoms, including valve defects, after at least six years of treatment. Since it was not possible to evaluate all patients after such a long period of therapy, the data cannot be conclusive, but this observation could be a starting point for further studies on the effects of long-term ERT, also on valve damage.

Among the valvulopathies, aortic insufficiency was the only one for which no regression was found despite ERT, rather its onset or worsening was reported at T1, with an incidence doubled at this time-point compared to diagnosis.

Valve defects were often found in association with other cardiac disorders. Given that GAG accumulation reduces the electrical conductivity of the myocardiocytes [16], reason why the electrocardiographic diagnostic criteria of ventricular hypertrophy are not respected, ventricular pseudohypertrophy was often associated with left valve defects, but not with heart failure. Heart failure, more frequent in MPS VI, was often associated with mitral stenosis and aortic steno-insufficiency.

The general clinical phenotype proved not to be strictly correlated with the cardiac one, in fact in some cases patients with attenuated phenotypes developed more severe heart damage than patients with severe phenotype. In this regard, an attempt was made to correlate cardiac damage with the genotype, or with the specific genomic variants, in homozigosis or hemizygosis. However, due to the limited data available in literature regarding the cardiological aspects of MPS patients harboring the same mutations as the patients enrolled in our study, in most cases it has not been possible to compare different patients with the same gene variant.

## Conclusions

In conclusion, our analysis confirms the wide presence of cardiopathies, at different extent, in the MPS population. Since cardiac pathology is the main cause of death in many MPS subtypes, it is necessary to raise awareness among cardiologists about the detection of early cardiac morpho-structural abnormalities. The encouraging data regarding the long-term effects of ERT, also on heart damage, underlines once again the importance of an early diagnosis and timely start of ERT, in order to prevent or slow down the organ damage resulting from GAGs storage, and to improve the quality of life of patients affected by these rare metabolic diseases.

## Methods

Sixty subjects affected by MPS were enrolled in a multicentre Italian study. Clinical, biochemical and molecular data was collected in a web-based platform shared among the different units. The diagnosis of MPS was accomplished by two-dimensional electrophoresis of urinary GAGs, while enzyme activity assays were performed in serum, leukocytes and/or skin fibroblasts. At the time of enrollment, molecular diagnosis was available for 50 subjects and it was recently described by Zanetti et al. [20]; in the same paper, the novel missense variants were evaluated in silico*,* for pathogenicity significance, by using different prediction tools [20].

Each patient underwent a multidisciplinary clinical follow-up, including cardiologic evaluation, examined in the present work. In this paper, we refer to three specific time-points: the time at diagnosis (indicated as TD), the closest time before the start of treatment (indicated as T0) and the time at enrolment in the study (indicated as T1). Being a treatment for MPS III and MPS IVB not yet available, for these patients we only refer to two time-points (TD and T1).

Cardiologic evaluation included physical examination, electrocardiogram and the echocardiogram. All patients were evaluated for ventricular hypertrophy, heart failure (NYHA Classification), valvulopathies (aortic and/or mitral and/or tricuspid stenosis, aortic and/or mitral and/or tricuspid regurgitation), heart rate and arrhythmias, conduction disorders, cardiomegaly and congenital cardiac abnormalities. Tricuspid valve regurgitation was considered as pathological, and distinguished from physiological regurgitation, for right ventricular pressure values higher than 20 mmHg. The frequency of each cardiac reported manifestation, its presence at the time of diagnosis or its appearance during clinical follow-up, and the coexistence in the same patient of several cardiologic disorders have been evaluated. In addition, we evaluated cardiac involvement in relation to age at start of ERT, by clustering patients in four groups: patients who had started therapy within the first year of age, between 1 and 3 years of age, between 3 and 10 years of age, and after 10 years of age. In these clusters we also distinguished between patients with mild or severe clinical phenotype.

As for the analysis of genetic variants, among patients for whom molecular diagnosis was available (a total of 50 subjects), only those presenting the pathogenic variants either in hemizygosis (for MPS II) or in homozygous condition, were considered, for a total of 31 subjects. We analyzed the possible impact of each individual variant on the cardiac phenotype. For this purpose, we reviewed any known association between genotype and cardiac involvement in the literature for comparison with the present cohort. We also tested whether variants associated with rapidly progressing phenotypes were unequivocally associated with more severe cardiac involvement.

## Data Availability

The data sets, including redacted study protocol, redacted statistical analysis plan, and individual participants' data supporting the results reported in this article, will be made available within 3 months from initial request, to researchers who provide a methodologically sound proposal. The data will be provided after its deidentification in compliance with applicable privacy laws, data protection and requirements for consent and anonymization.

## Abbreviations

MPS: Mucopolysaccharidosis
GAGs: Glycosaminoglycans
ERT: Enzyme replacement therapy
HSCT: Hematopoietic stem cell transplantation
HO: Homozygosis
HE: Hemyzygosis
TD: Time at diagnosis
T0: The closest time before treatment
T1: Time at enrolment in the study
NA: Not available
